# Highly Fluorescent Dyes Containing Conformationally Restrained Pyrazolylpyrene (Pyrazoolympicene) Chromophore

**DOI:** 10.3390/molecules27041272

**Published:** 2022-02-14

**Authors:** Anna Wrona-Piotrowicz, Anna Makal, Janusz Zakrzewski

**Affiliations:** 1 Department of Organic Chemistry, Faculty of Chemistry, University of Lodz, Tamka 12, 91-403 Lodz, Poland; janusz.zakrzewski@chemia.uni.lodz.pl; 2 Biological and Chemical Research Center, Faculty of Chemistry, University of Warsaw, Żwirki i Wigury 101, 02-089 Warsaw, Poland; am.makal@uw.edu.pl

**Keywords:** pyrazoolympicene, fluorescence, DFT calculations

## Abstract

The triflic-acid-promoted cyclization of 1-phenyl-3-(pyren-1-yl)-1*H*-pyrazole-4-carbaldehyde afforded a mixture of 9-phenyl-7,9-dihydropyreno (10,1-*fg*)indazole and 9-phenylpyreno(10,1-*fg*)indazole-7(9*H*)-one, readily separable by column chromatography. Both products contained a rigid six-ringed pyrazoolympicene backbone and exhibited bright fluorescence in chloroform solution and a weak fluorescence in the solid state. DFT and TD DFT calculations revealed that the lowest excited state (S_1_) of these compounds is populated via HOMO →LUMO π-π * transition. Furthermore, the synthesized compounds behaved as weak bases and their emission spectra showed substantial changes upon protonation. Therefore, they may be of interest for sensing of strongly acidic fluorophore environments.

## 1. Introduction

Pyrene is one of the most interesting fluorophores, exhibiting intense and environment-sensitive emission [1,2], aggregation-induced emission (AIE) [3] and capable of forming emissive excimers and exciplexes [4,5,6]. Moreover, it can also be easily functionalized [7,8,9]. Due to these properties, pyrene has been the subject of interest in numerous studies aimed at its modification to obtain useful fluorophores for practical applications in various branches of science, ranging from optoelectronics to biology and medicine [4,7,10,11,12,13,14]. Pyrazoles are another class of small molecule fluorophores exhibiting intense and tunable emission, thoroughly studied in recent years [15,16,17,18,19,20]. Therefore, it seemed interesting to synthesize molecules with conjugated pyrene-pyrazole cores and to study their luminescent properties.

**Figure 1:**
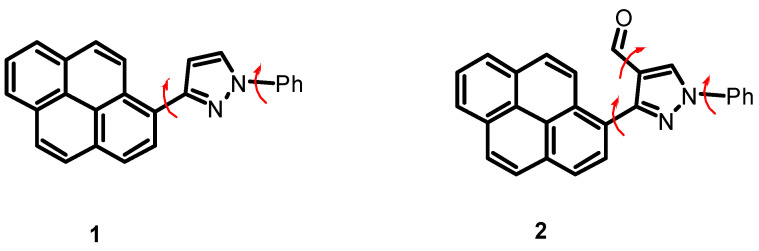
Molecular structures of pyrazolylpyrenes **1** and **2**. Arrows indicate possible rotations around single bonds.

We previously reported the synthesis and fluorescent properties of pyrazolylpyrenes **1** and **2** (Figure 1) [21,22].

However, both molecules are conformationally labile and may adopt nonplanar geometries, which may decrease their emission efficiencies (their emission quantum yields in chloroform are 0.69 and 0.26, respectively). Therefore, we became interested in the synthesis of conformationally restrained and planarized analogs of these compounds with the aim of batochromically shifting their emission maxima and improving their emission quantum yields. The bathochromic shift of the absorption and emission bands and the increase in the emission efficiency of the fluorophore resulting from the structural prevention of intramolecular rotations have already been demonstrated for various classes of dye [23,24,25]

**Scheme 1:**
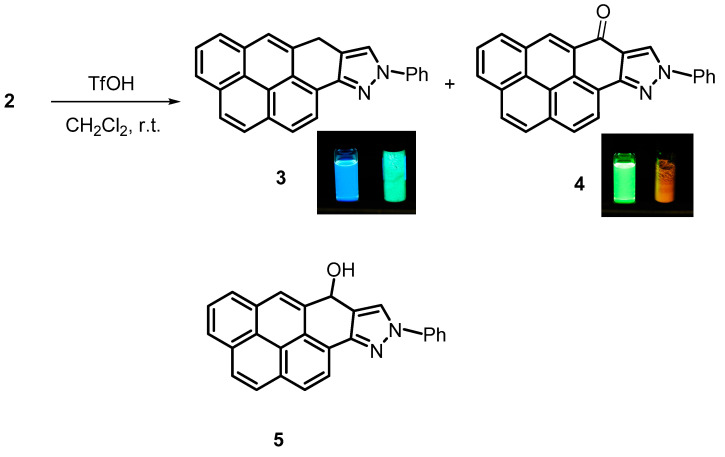
Triflic acid-promoted cyclization of **2**. Insets show the fluorescence of **3** and **4** in chloroform solutions (left vial on both pictures) and solid samples (right vial on both pictures) illuminated with a UV light.

Herein, we disclose the synthesis of dyes **3** and **4** (Scheme 1), along with the study of their structures and optical properties, in comparison to those of **1** and **2**.

## 2. Results and Discussion

### 2.1. Synthesis of ***3*** and ***4***

**Figure 2:**
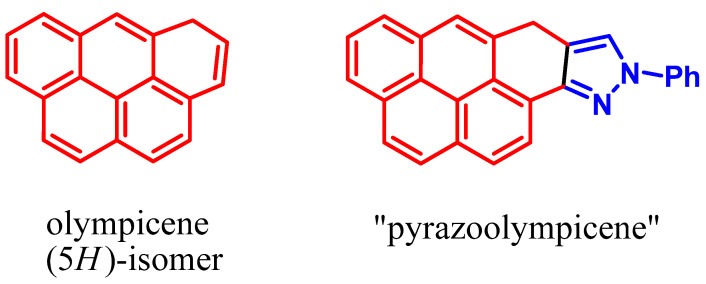
Structures of (*5H*)-olympicene [27] and “pyrazolympicene”.

Inspired by the Klumpp’s work on the superacid-promoted cyclization of naphthyl pyrazolecarbaldehyde [26], we investigated the possibility of performing a similar cyclization with compound **2**. Pleasingly, we found that the addition of an excess of trifluoromethanesulfonic (triflic) acid to a solution of this compound in dichloromethane at room temperature resulted in the rapid formation of fluorescent cyclization products, 9-phenyl-7,9-dihydropyreno (10,1-*fg*)indazole, **3** and 9-phenylpyreno(10,1-*fg*)indazole-7(*9H*)-one, **4**, readily separable by column chromatography. Both compounds contain a “pyrazoolympicene” six-ringed system (Figure 2).

**Figure 3:**
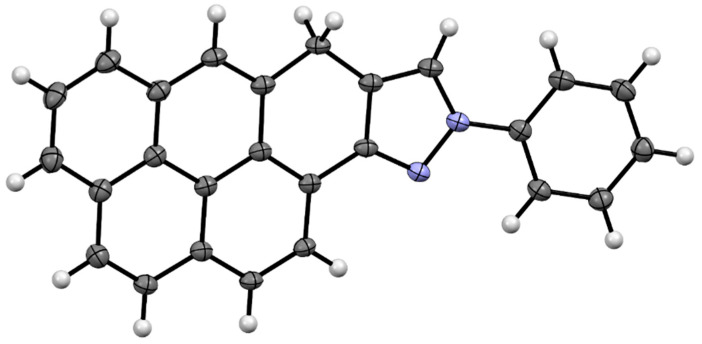
Molecular structure of compound **3**, determined by X-ray diffraction.

The structures of **3** and **4** were confirmed by spectroscopic methods. That of **3** was confirmed by single crystal X-ray diffraction (Figure 3).

**Figure 4:**
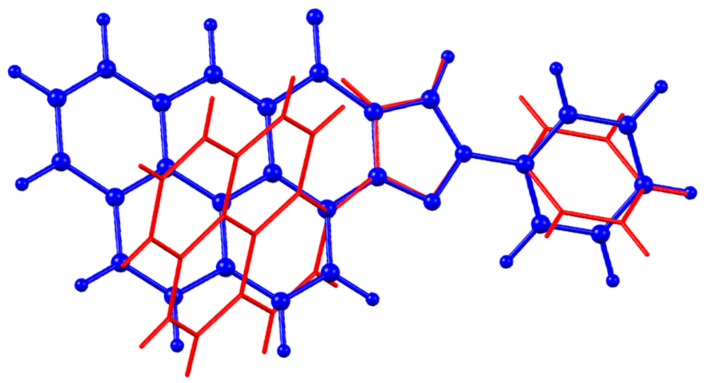
Overlay of the X-ray diffraction-derived structures of **3** (blue) and **1** (red).

As expected, unlike the highly twisted molecules of **1** [21], the molecules of **3** were found to be planar (Figure 4 and Supplementary Information).

The mechanism proposed by Klumpp [26] suggests that compounds **3** and **4** may be formed by the disproportionation of alcohol, **5**, which is the presumed initial cyclization product, and thus obtained in equivalent amounts. However, we found that **3** tended to undergo partial oxidation to **4** on workup. As a result, the isolated yields of **3** and **4** were different and not strictly reproducible (15–25% for **3** and 70–80% for **4**).

### 2.2. Photophysical Properties of ***1***–***4***

#### 2.2.1. UV/VIS Absorption and Emission Spectra in Solution

**Figure 5:**
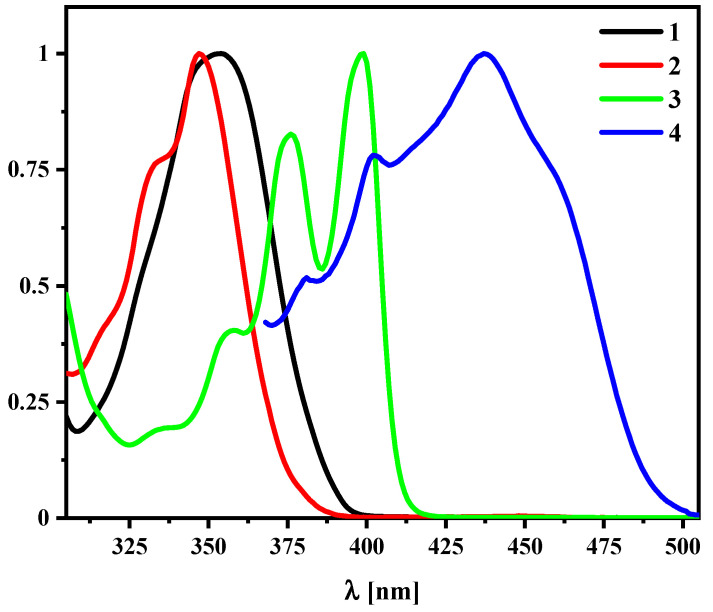

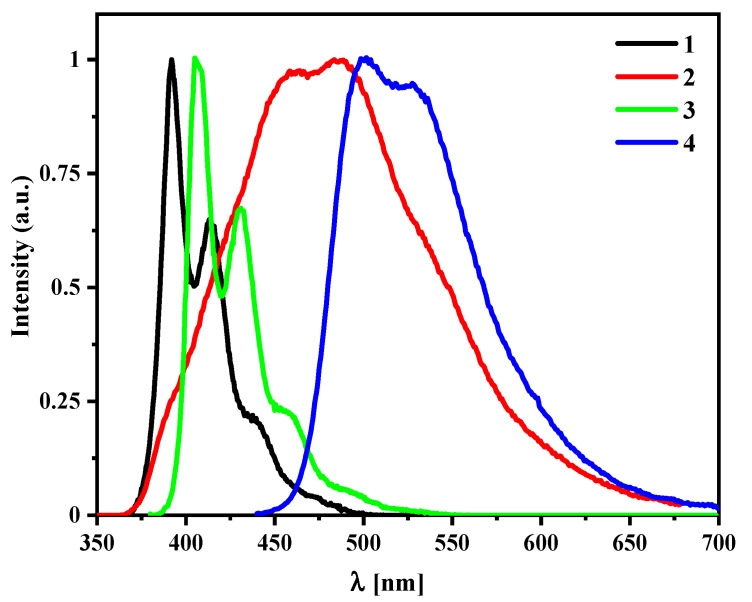
Electronic absorption (**top**) and emission (**bottom**) spectra of compounds **1**–**4**. Excitation at the maxima of absorption.

The electronic absorption and emission spectra of compounds **1**–**4** in chloroform are shown in Figure 5 and the corresponding spectroscopic data are presented in Table 1.

It can be seen that the absorption bands of conformationally restrained compounds **3** and **4** are bathochromically shifted relative to the bands of their conformationally labile counterparts, **1** and **2**. We noted that in contrast to the broad, structureless absorption band of compound **1**, the red-shifted absorption band of compound **3** showed a well-resolved vibronic structure. On the other hand, the emission spectra of both compounds displayed structured bands at highly similar wavelengths, which suggests that their emitting states had similar geometries, and thus compound **1** was planarized upon photoexcitation. Moreover, the comparison of the emission quantum yields of **1** and **3** (0.69 and 0.77, respectively) indicated that the rotation around the C-C bond linking the pyrazole and pyrene units cannot be considered as an efficient nonradiative excited state deactivation channel. Therefore, it can be assumed that the decrease in the emission efficiency of compound **2** can be related to deactivation via the rotation of the aldehyde group. The higher emission quantum yield of compound **4** (0.94) can be attributed to the restriction of this rotation.

#### 2.2.2. Solid State Fluorescence of **3** and **4**

**Figure 6:**
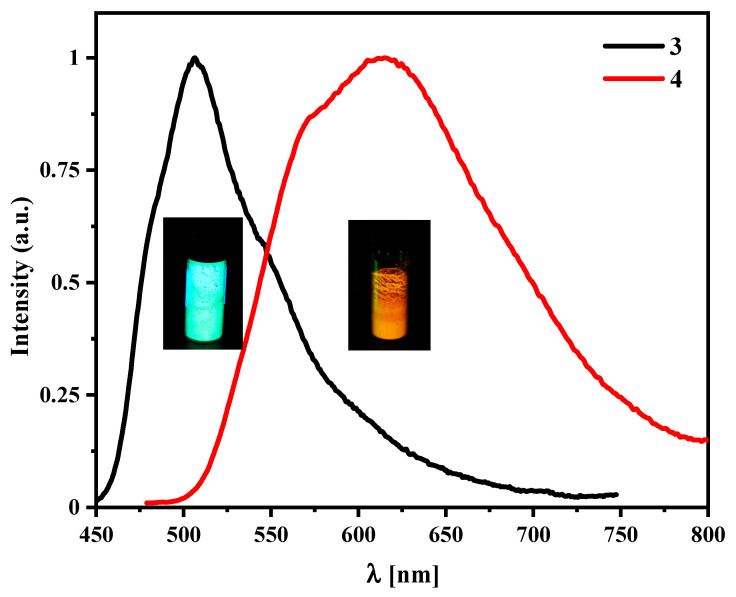
Normalized solid-state emission spectra of compounds **3** and **4** (excitation at 390 nm for **3** and 420 nm for **4**).

Microcrystalline solid samples of **3** and **4** displayed a weak fluorescence (quantum yields of 0.045 and 0.08, respectively) (Figure 6). In comparison to the emission bands observed in solution the bands of solid samples were broad, structureless and red shifted (maxima at 506 nm and 615 nm, respectively), which suggests that they might have originated from aggregates or excimers.

**Figure 7:**
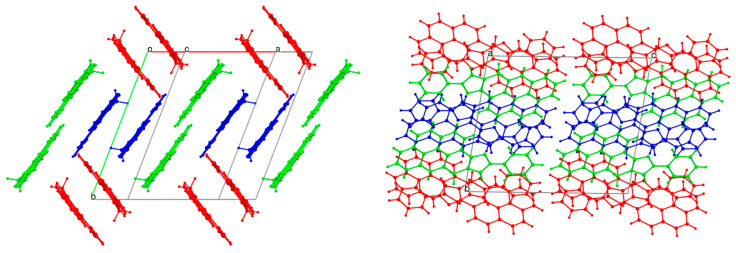
Crystal packing of compound **3** in selected directions (three crystallographically independent molecules are highlighted in colors). Left: View in the direction parallel to the pyrene planes of all molecules, showing π · π stacked dimers; Right: View along [100] showing the layers separation along [001]. More details are provided in Supplementary Information.

The presence of π···π stacked dimers in the crystal of **3** was confirmed by X-ray diffraction data (Figure 7).

### 2.3. Theoretical Calculations

For an in-depth understanding of the molecular structures and electronic transitions leading to the lowest-energy singlet-excited states of compounds **3** and **4**, we performed density functional theory (DFT) and time-dependent density functional theory (TD-DFT) calculations using Gaussian16 package and B3PW91/6-311++G(d,p) basis set. The calculations were carried out for isolated molecules with the solvent (chloroform) simulated in polarizable continuous model (PCM) approximation. The DFT calculations were performed using resources from WCSS (grant 115).

#### 2.3.1. Geometry Optimizations

The optimized structures of **3** and **4**, in their ground (S_0_) and in their first excited state (S_1_), are shown in Figure S2 (Supplementary Information). The significant interatomic distances, in comparison to the X-ray diffraction data obtained for **3**, are presented in Table S2 (Supplementary Information).

In the S_0_ state, the six-ringed system was planar in both compounds. In compound **4**, the phenyl ring was found to be twisted out by an angle of approximately 30°, but the twist disappeared in the S_1_ state (Figure S2d).

**Figure 8:**
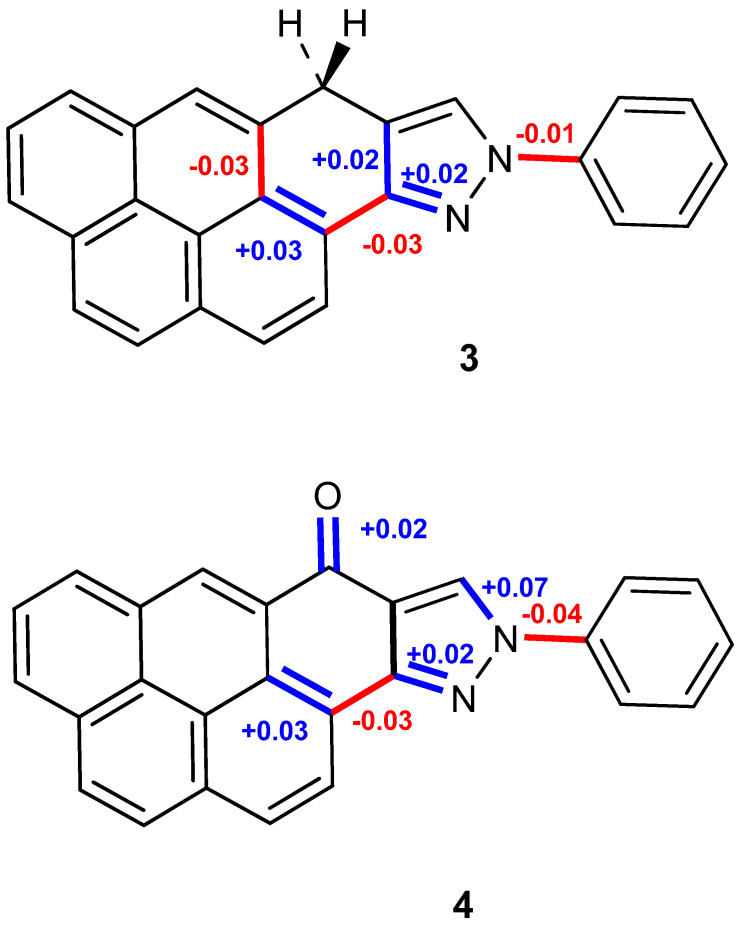
Significant bond length changes in **3** and **4** upon S_0_→S_1_ excitation. Contracted bonds are represented in red and elongated bonds in blue. Bond length changes are expressed in Å.

The changes in the bond lengths upon excitation are schematically represented in Figure 8. There was a tendency towards the equalization of the bond lengths between the formally single and double bonds, indicating more efficient electronic conjugation along the π-backbone in the S_1_ state.

#### 2.3.2. Molecular Orbitals

**Figure 9:**
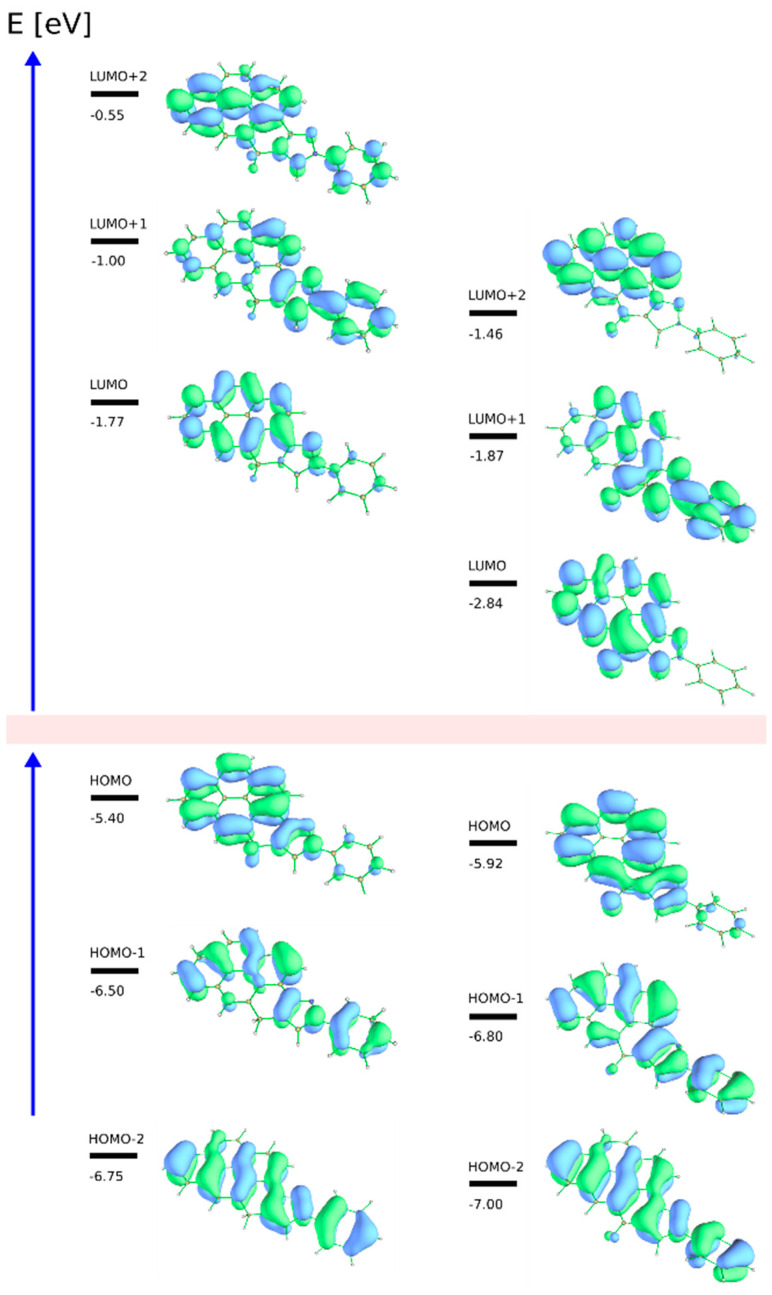
DFT-derived shapes of the molecular orbitals of **3** (left) and **4** (right) and their energies in chloroform solution. Iso-surfaces at 0.024 a.u.

The main molecular orbitals of compounds **3** and **4** are shown in Figure 9.

We found that the highest occupied molecular orbital (HOMO) and lowest unoccupied molecular orbitals (LUMO) of **3** and **4** were mostly localized on the pyrene fragment, with a certain degree of extension on the pyrazole ring and (for **4**) on the carbonyl group. The energies of these orbitals and the HOMO-LUMO gap of compound **3** were comparable to those of pyrene (HOMO: −5.33 eV, LUMO: −1.50 eV, HOMO-LUMO gap: 3.83 eV (in gas phase) [28]. On the other hand, the HOMO of **4** was stabilized by ~0.6 eV, while the stabilization of LUMO was stronger (~1.3 eV). As a result, the HOMO-LUMO gap of this compound (3.08 eV) was significantly lower than that of **3** (3.63 eV).

#### 2.3.3. Electronic Transitions

The nature, energies and oscillator strengths of the lowest energy (S_0_→S_1_) transitions of compounds **1–4** are presented in Table 2.

**Figure 10:**
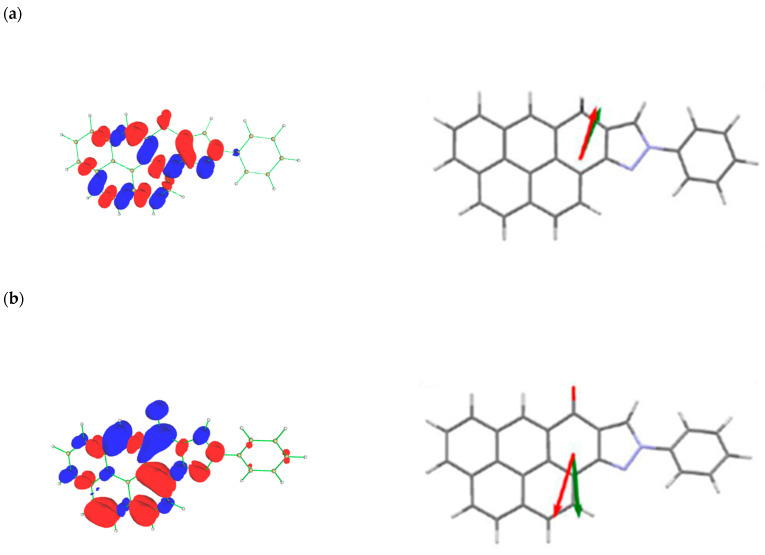
(**Left**) The difference in electron density distribution (regions with increased electron concentration indicated in blue, and decreased electron concentration in red) [29] for the S_0_→S_1_ excitation and (**right**) molecular dipole moments for the S_0_ (green) and S_1_ (red) states of compounds **3** (**a**) and **4** (**b**). Iso-surfaces at ± 0.001 eÅ^−3^.

The first singlet electronic excited states of **1**, **3** and **4** resulted from almost pure HOMO → LUMO (π→π*) transitions, while for **2**, the theory predicts an admixture from HOMO → LUMO + 1 transition. These transitions are associated with charge reorganization within pyrene-pyrazole moieties. In the case of **3** and **4**, they practically do not cause any change in molecular dipole moment (only minor changes in dipole orientation) (Figure 10). The values of the dipole moments calculated for compound **3** in chloroform are 3.09 D in the ground (S_0_) state and 3.06 D in the excited (S_1_) state, and the values for **4** are 2.70 D and 2.84 D, respectively.

**Figure 11:**
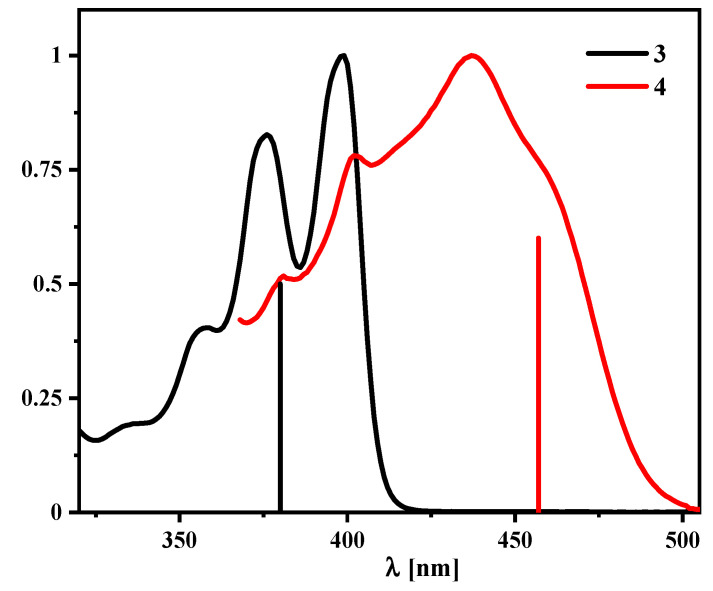
Comparison of calculated (vertical lines) and experimental electronic absorption spectra of **3** and **4**.

The calculations for **3** predicted an absorption spectrum with a maximum at 380 nm. The experimental spectrum of this compound contains vibronic bands at 357, 376 and 399 nm. The predicted maximum is close to the center of the mass of the experimental spectrum (Figure 11), although its vibrational structure could not be reproduced at this level of theory.

For compound **4**, the theory predicts the lowest energy band with a maximum at 457 nm, whereas the experimental spectrum contains a broad band with poorly resolved vibronic structure, with a maximum at 432 nm and a shoulder at ~460 nm (Figure 11).

### 2.4. Acidity Sensing

**Figure 12:**
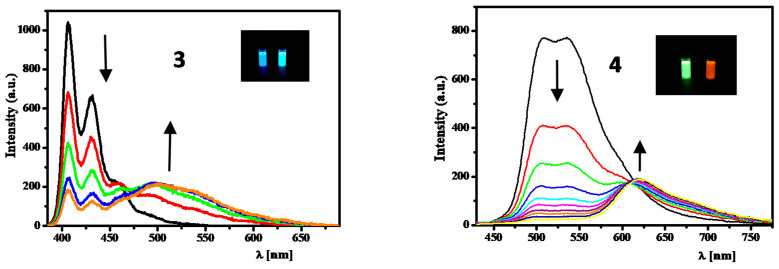
Emission spectra of dichloromethane solutions of **3** and **4** (c = 5 µM) titrated with TFA at a concentrations of 0, 20, 40, 60 and 80 mM for **3** and 0, 20, 40, 60, 80, 100, 120, 140 and 160 mM for **4** (excitation at 376 nm for **3** and 400 nm for **4**). Insets show the emission colors, under UV light, in the absence of TFA and in the presence of excess TFA (left and right, respectively).

We expected that **3** and **4** would be endowed with weak basic properties by the 1-phenylpyrazole fragment (pKa 0.43–0.44 [30,31]); therefore, we investigated the photophysical properties of these compounds in acidic media. The effect of the addition of trifluoroacetic acid (TFA) on the emission spectra of **3** and **4** in dichloromethane is shown in Figure 12.

As can be seen, the addition of TFA led to the gradual disappearance of the bands of **3** and **4** while resulting in the appearance of broad, red-shifted bands, which can be assigned to their protonated forms.

The above findings indicate that compounds **3** and **4** can act as fluorescent sensors of strongly acidic fluorophore environments (during the titration, the acidity expressed by *Ho* changed in the range 2.1 ÷ 0.3 for 3 and 2.1 ÷ −0.4 for 4 [32]). It is worthy of note that only a few examples of these sensors have been reported so far [6,33].

## 3. Conclusions

We developed a simple method to synthesize a novel polycyclic *N*-heterocyclic fluorophore system, pyrazoolympicene, (7,9-dihydropyreno (10,1-*fg*) indazole), which exhibits intense fluorescence and may be used for sensing strongly acidic environments. We expect that the photophysical properties of this system can be tuned by various structural modifications of the reactive 1-phenylpyrazole fragment and the carbonyl group.

## 4. Materials and Methods

All reagents and solvents were purchased from Sigma-Aldrich and used without further purification. Compound **2** was prepared as described in [22]. Column chromatography was carried out on silica gel 60 (0.040–0.063 mm, 230–400 mesh, Fluka). ^1^H and ^13^C NMR spectra were recorded at room temperature (291 K) in CDCl_3_ on a Bruker ARX 600 MHz (600 MHz for ^1^H and 151 MHz for ^13^C). Chemical shifts are in ppm and coupling constants in Hz. Fourier transform-infrared (FT-IR) spectroscopy data were obtained from Thermo Nicolet Nexus FT-IR. The spectra were recorded from an accumulation of 32 scans in the range of 4000~400 cm^−1^. Mass spectra were recorded on Varian 500-MS LC Ion Trap. Elemental analyses were performed in the Microanalytical Laboratory of the Faculty of Chemistry, University of Lodz.

### 4.1. Synthesis of ***3*** and ***4***

Triflic acid (176 µL, 2 mmol) was added to a solution of **2** (372 mg, 1 mmol) in dichloromethane (10 mL). After stirring for 1 h, the reaction mixture was poured into water (100 mL) and extracted several times with dichloromethane. The combined extracts were dried over anhydrous Na_2_SO_4_ and evaporated to dryness. The products were separated by column chromatography on silica gel (dichloromethane as eluent).

9-phenyl-7,9-dihydropyreno(10,1-*fg*)indazole (**3**). Yellow powder (54–89 mg, 15–25%); mp = 347–348 °C; ^1^H NMR (600 MHz, CDCl_3_) δ 8.72 (d, *J* = 7.8 Hz, 1H), 8.21 (d, *J* = 7.8 Hz, 1H), 8.08 (d, *J* = 7.8 Hz, 1H), 8.04 (d, *J* = 7.8 Hz, 1H), 8.02 (q, *J* = 9.0 Hz, 2H), 7.95 (m, 2H), 7.86 (s, 1H), 7.84 (m, 2H), 7.50 (t, *J* = 7.8 Hz, 2H), 7.31 (t, *J* = 7.5 Hz, 1H), 4.66 (s, 2H); ^13^C{^1^H} NMR (151 MHz, CDCl_3_) δ 148.9, 140.4, 131.6, 131.5, 131.2, 131.1, 129.45, 127.7, 127.0, 126.7, 126.2, 126.17,126.15, 125.8, 125.1, 124.4, 124.1, 124.0, 123.9, 119.6, 118.9, 116.6, 26.5; MS (ESI): m/z: 357 (M)+, 379 (M + Na)+; Anal. calcd. for C_26_H_16_N_2_: C, 87.62; H, 4.52; found: C, 87.48; H, 4.67.

9-phenylpyreno(10,1-*fg*)indazole-7(*9H*)-one (**4**). Orange powder (260–297 mg, 70–80%); mp = 336–337 °C; 1H NMR (600 MHz, CDCl3) δ 9.44 (s, 1H), 9.00 (d, *J* = 7.8 Hz, 1H), 8.80 (s, 1H) 8.54 (d, *J* = 7.8 Hz, 1H), 8.40 (d, *J* = 7.8 Hz, 1H), 8.37 (d, *J* = 7.8 Hz, 1H), 8.18 (q, *J* = 9.0 Hz, 2H), 8.14 (t, *J* = 7.5 Hz, 1H), 7.97 (m, 2H), 7.59 (m, 2H) 7.45 (t, *J* = 7.5 Hz, 1H); IR (KBr, cm^−1^) 1659,5 (C=O); MS (ESI): m/z: 371 (M)+, 393 (M + Na)+; Anal. calcd. for C_26_H_14_N_2_O: C, 84.31; H, 3.81; found: C, 84.22; H, 3.76.

All spectra are in Supplementary Materials.

### 4.2. UV/Vis Measurements

The electronic absorption spectra were obtained using a PerkinElmer Lambda 45 UV/VIS spectrometer, and the corrected emission spectra using a PerkinElmer LS-55 fluorescence spectrometer. The emission quantum yields were determined using a solution of quinine sulfate in 0.5 M sulfuric acid as a reference (ΦF = 0.546) [34].

### 4.3. X-ray Diffraction Measurment

The X-ray intensity data were measured on an Agilent Supernova 4 circle diffractometer system equipped with a copper (CuKα) microsource and an Atlas CCD detector. The data were collected and integrated with CrysAlis171 software (version 1.171.38.43d). The data were corrected for absorption effects using the multi-scan method CrysAlis171 software (version 1.171.38.43d).

The low temperature of the sample was maintained by keeping it in a cold nitrogen stream, using Oxford Cryosystems cooling devices.

The structure was solved by direct methods using SXELXS [35] and refined by full-matrix least squares procedure with SHELXL [35] within an OLEX2 [36] graphical interface. Figures were produced with Mercury_3.10 [37] software.

All H atoms were visible in the residual density map, but were added geometrically and refined mostly in riding approximation.

Detailed information about the data processing, structure solution and refinement are presented in Table S1.

## Figures and Tables

**Table 1:** Photophysical data of compounds **1–4**.

Compound	Absorption (nm)/ε_max_ (M^−1^ cm^−1^)	Emission (nm)	Stokes Shift (nm/cm^−1^)	Quantum Yield ΦF
**1**	352/45,570/ *	392, 415, 438 (sh) *	40/2900	0.69 *
**2**	332/25,240/, 347/32,640/ **	449 **	102/6547	0.26 **
**3**	357/23,100/, 376/47,240/, 399 (57,140)	406, 432, 459	7/432	0.77
**4**	381/6480/, 402/9770/, 437/12,510/	503, 532	66/3003	0.94

* Data taken from [21] ** Data taken from [22].

**Table 2:** TD-DFT calculated electronic transitions involved in the formation of lowest-energy singlet excited states (S_0_→S_1_) of compounds **1–4** in chloroform, their energies, wavelengths and oscillator strengths (f).

Compound	Contributing Orbitals (Contribution)	ΔE [eV]	λ_calc_ (nm)	f
**1** *	H → L (0.93)	3.41	363.5	0.626
**2** **	H → L (0.67) H → L + 1 (0.30)	3.31	375.0	0.236
**3**	H → L (0.96)	3.26	380.2	0.806
**4**	H → L (0.96)	2.71	457.1	0.29

* Data taken from [21] ** Data taken from [22].

## Data Availability

The structure of **3** has been deposited with CCDC, deposition number 1935802.

## Supplementary Materials

The following supporting information can be downloaded. Figure S1: Molecular structure of compound 3 determined by X-ray diffraction; Figure S2: The overlay of the molecular structures of **3** and **4**; Figure S3: ^1^H NMR spectrum of compound **3**; Figure S4: ^13^C NMR spectrum of compound **3**; Figure S5: IR (KBr) spectrum of compound **3**; Figure S6: ESI MS spectrum of compound **3**; Figure S7: ^1^H NMR spectrum of compound **4**; Figure S8: IR (KBr) spectrum of compound **4**; Figure S9: ESI MS spectrum of compound **4**; Table S1: X-ray diffraction data for **3**; Table S2: Comparison of experimental and theoretical geometries of **3** and **4**.

## Sample Availability

Samples of the compounds are available from the authors.

## References

[1] Winnik F.M. (1993). Photophysics of preassociated pyrenes in aqueous polymer solutions and in other organized media. Chem. Rev..

[2] D’Abramo M., Aschi M., Amadei A. (2015). Theoretical calculation of the pyrene emission properties in different solvents. Chem. Phys. Lett..

[3] Islam M.M., Hu Z., Wang Q., Redshaw C., Feng X. (2019). Pyrene-based aggregation-induced emission luminogens and their applications. Mater. Chem. Front..

[4] Duhamel J. (2012). New Insights in the Study of Pyrene Excimer Fluorescence to Characterize Macromolecules and their Supramolecular Assemblies in Solution. Langmuir.

[5] Karuppannan S., Chambron J.-C. (2011). Supramolecular Chemical Sensors Based on Pyrene Monomer–Excimer Dual Luminescence. Chem. Asian J..

[6] Liu T., Huang Z., Feng R., Ou Z., Wang S., Yang L. (2020). An intermolecular pyrene excimer-based ratiometric fluorescent probes for extremely acidic pH and its applications. Dyes Pigm..

[7] Figueira-Duarte T.M., Müllen K. (2011). Pyrene-Based Materials for Organic Electronics. Chem. Rev..

[8] Feng X., Hu J.-Y., Redshaw C., Yamato T. (2016). Functionalization of Pyrene To Prepare Luminescent Materials-Typical Examples of Synthetic Methodology. Chem. Eur. J..

[9] Casas-Solvas J.M., Howgego J.D., Davis A.P. (2014). Synthesis of substituted pyrenes by indirect methods. Org. Biomol. Chem..

[10] Kinik F.P., Ortega-Guerrero A., Ongari D., Ireland C.P., Smit B. (2021). Pyrene-based metal organic frameworks: From synthesis to applications. Chem. Soc. Rev..

[11] Hirai Y., Laize-Générat L., Wrona-Piotrowicz A., Zakrzewski J., Makal A., Brosseau A. (2021). Multi-Directional Mechanofluorochromism of Acetyl Pyrenes and Pyrenyl Ynones. ChemPhysChem.

[12] Bains G., Patel A.B., Narayanaswami V. (2011). Pyrene: A Probe to Study Protein Conformation and Conformational Changes. Molecules.

[13] Ayyavoo K., Velusamy P. (2021). Pyrene based materials as fluorescent probes in chemical and biological fields. New J. Chem..

[14] Krasheninina O.A., Novopashina D.S., Apartsin E.K., Venyaminova A.G. (2017). Recent Advances in Nucleic Acid Targeting Probes and Supramolecular Constructs Based on Pyrene-Modified Oligonucleotides. Molecules.

[15] Tigreros A., Portilla J. (2020). Recent progress in chemosensors based on pyrazole derivatives. RSC Adv..

[16] Willy B., Müller T.J.J. (2011). Rapid One-Pot, Four-Step Synthesis of Highly Fluorescent 1,3,4,5-Tetrasubstituted Pyrazoles. Org. Lett..

[17] Willy B., Müller T.J.J. (2008). Regioselective Three-Component Synthesis of Highly Fluorescent 1,3,5-Trisubstituted Pyrazoles. Eur. J. Org. Chem..

[18] Mukherjee S., Salini P.S., Srinivasan A., Peruncheralathan S. (2015). AIEE phenomenon: Tetraaryl vs. triaryl pyrazoles. Chem. Comm..

[19] Götzinger A.C., Theßeling F.A., Hoppe C., Müller T.J.J. (2016). One-Pot Coupling–Coupling–Cyclocondensation Synthesis of Fluorescent Pyrazoles. J. Org. Chem..

[20] Amoah C., Obuah C., Ainooson M.K., Muller A. (2021). Synthesis, characterization and fluorescent properties of ferrocenyl pyrazole and triazole ligands and their palladium complexes. J. Organomet. Chem..

[21] Flamholc R., Zakrzewski J., Makal A., Brosseau A., Métivier R. (2016). Synthesis, regioselective aerobic Pd(II)-catalyzed C–H bond alkenylation and the photophysical properties of pyrenylphenylpyrazoles. Photochem. Photobiol. Sci..

[22] Flamholc R., Wrona-Piotrowicz A., Makal A., Zakrzewski J. (2018). Pyrenylpyrazole-based donor/acceptor fluorescent dyes: Synthesis and photophysical properties. Dye. Pigm..

[23] Matikonda S.S., Hammersley G., Kumari N., Lennart Grabenhorst L., Glembockyte V., Tinnefeld P., Ivanic J., Levitus M., Schnermann M.J. (2020). Impact of Cyanine Conformational Restraint in the Near-Infrared Range. J. Org. Chem..

[24] Ren T., Xu W., Jin F., Cheng D., Zhang L., Yuan L., Zhang X. (2017). Rational Engineering of Bioinspired Anthocyanidin Fluorophores with Excellent Two-Photon Properties for Sensing and Imaging. Anal. Chem..

[25] Bell J.D., Harkiss A.H., Nobis D., Malcolm E., Knuhtsen A., Wellaway C.R., Jamieson A.G., Magennis S.W., Sutherland A. (2020). Conformationally rigid pyrazoloquinazoline α-amino acids: One- and two-photon induced fluorescence. Chem. Commun..

[26] Klumpp D.A., Kindelin P.J., Li A. (2005). Superacid-promoted reactions of pyrazolecarboxaldehydes and the role of dicationic electrophiles. Tetrahedron Lett..

[27] Mistry A., Moreton B., Schuler B., Mohn F., Meyer G., Gross L. (2015). The Synthesis and STM/AFM Imaging of ‘Olympicene’ Benzo[cd]pyrenes. Chem. Eur. J..

[28] Örücü H., Acar N. (2015). Effects of substituent groups and solvent media on Pyrene in ground and excited states: A DFT and TDDFT study. Comput. Theor. Chem..

[29] Lu T., Chen F. (2012). Multiwfn: A multifunctional wavefunction analyzer. J. Comput. Chem..

[30] El Ghomari M.J., Mokhlisse R., Laurence C., Le Questel J.Y., Berthelot M. (1997). Basicity of azoles: Complexes of diiodine with imidazoles, pyrazoles and triazoles. J. Phys. Org. Chem..

[31] Marín-Luna M., Alkorta I., Elguero J. (2015). A theoretical study of the gas phase (proton affinity) and aqueous (pKa) basicity of a series of 150 pyrazoles. New J. Chem..

[32] Suslova E.E., Ovchenkova E.N., Lomova T.N. (2014). The Hammett acidity function H0 in trifluoroacetic acid–dichloromethane mixtures. Tetrahedron Lett..

[33] Shih I.-C., Yeh Y.-S., Wu I.-C., Chen Y.-H., Shen J.-Y., Chen Y.-A. (2015). A New Series of Fluorescent Indicators for Super Acids. Photochem. Photobiol..

[34] Brouwer A.M. (2011). Standards for photoluminescence quantum yield measurements in solution (IUPAC Technical Report). Pure Appl. Chem..

[35] Sheldrick G.M. (2008). A short history of SHELX. Acta Cryst..

[36] Dolomanov O.V., Bourhis L.J., Gildea R.J., Howard J.A.K., Puschmann H. (2009). OLEX2: A complete structure solution, refinement and analysis program. J. Appl. Cryst..

[37] Macrae C.F., Edgington P.R., McCabe P., Pidcock E., Shields G.P., Taylor R., Towler M., van de Streek J. (2006). Mercury: Visualization and analysis of crystal structures. J. Appl. Cryst..

