# Supplementary material for: Highly Fluorescent Dyes Containing Conformationally Restrained Pyrazolylpyrene (Pyrazoolympicene) Chromophore
Source: Molecules. 2022 Feb 14;27(4):1272. doi: 10.3390/molecules27041272 (PMC8875643; doi:10.3390/molecules27041272)
Supplement: Supplementary file 1 [file molecules-27-01272-s001.zip › molecules-1573408-supplementary.pdf]

Supplementary Information

Highly fluorescent dyes containing conformationally restrained pyrazolylpyrene (pyrazoolympicene) chromophore

Anna Wrona-Piotrowicz, Anna Makal, Janusz Zakrzewski

1. X-ray diffraction data for **3**……………………………………………………….……..2
2. Geometry optimization of **3** and **4.** Comparison of calculated and experimental data……...4
3. ^{1}H and ^{13}C NMR, IR and ESI MS spectra of **3** and **4**………………...……………………...6

1. X-ray diffraction data for 3

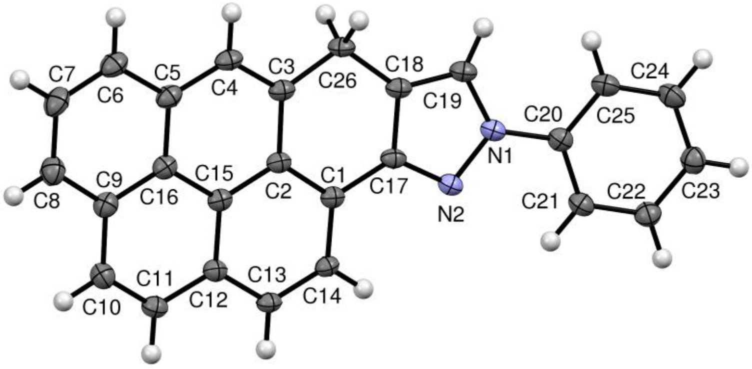

Figure S1. Molecular structure of compound **3** determined by X-ray diffraction.

Crystals of **3** suitable for the X ray diffraction study were grown from layered dichloromethane- pentane. The X-ray intensity data were measured on Agilent Supernova 4 circle diffractometer system equipped with copper (CuKα) microsource and Atlas CCD detector. The data were collected and integrated with CrysAlis171[10] software. Data were corrected for absorption effects using the multi-scan method (SCALE3 ABSPACK[10]).
Low temperature of the sample was maintained by keeping it in the cold nitrogen stream, using Oxford Cryosystems cooling devices.
The structure was solved by direct methods using SXELXS[vi] and refined by full-matrix least squares procedure with SHELXL[vi] within OLEX2[vii] graphical interface. Figures were produced with Mercury_3.10[ix] software.
All H atoms were visible in the residual density map, but were added geometrically and refined mostly in riding approximation.
Notably, compound **3** crystallized with three independent molecules in the crystallographic asymmetric unit. These molecules presented very similar molecular geometry but formed three distinct π-stacked dimers in the crystal structure (depicted in red, blue and green accordingly in Figure 7 of the main text).
Detailed information about the data processing, structure solution and refinement is presented in Table S1. The structure has been deposited with CCDC, deposition number 1935802.

Table S1. X-ray diffraction data for **3**

**Compound**	**3**
Empirical formula	$C_{26} H_{12} N_2$
Formula weight	356.41
Crystal system	triclinic
Space group	P -1
a/Å	11.8406(3)
b/Å	14.2522(4)
c/Å	15.9960(4)
α/°	97.910(2)
β/°	91.947(2)
γ/°	109.443(2)
Volume/$Å^3$	2512.01(12)
Z / Z’	6 / 3
μ/mm^{-1}	0.643
Max. transmission	1.00000
Min. transmission	0.89380
Absorption correction	multi-scan
Crystal color	yellow
ρ_{calc}mg/mm^3	1.414
Crystal habit	plate
F(000)	1116
Crystal size/mm	0.22
	0.19
	0.05
R_{int}	0.0250
R_{sigma}	0.0209
Index ranges	
h	14
	-14
k	17
	-17
l	20
	-20
Reflections collected	41398
2Θ range for data collection	76.504
	3.301
Temperature/K	100.1(1)
X-ray wavelength/Å	1.54184
Independent reflections I > 2 σ(I)	8745
Independent reflections	10482
Largest diff. peak/hole /e $Å^{-3}$	0.278
	-0.190
Goodness-of-fit on F^2	1.043
Parameters	757
Data	10482
Restraints	0
R1 all data	0.0463
R1 [I>=2σ (I)]	0.0377
wR2 [I>=2σ (I)]	0.1052
wR2 all data	0.1125

Z’ - indicates the number of crystallographically independent molecules

2. Geometry optimization of 3 and 4. Comparison of calculated and experimental data

(a)

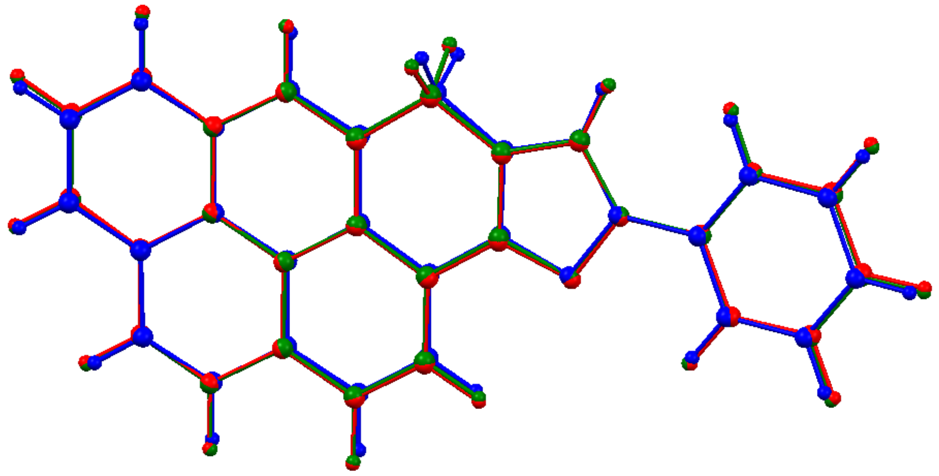

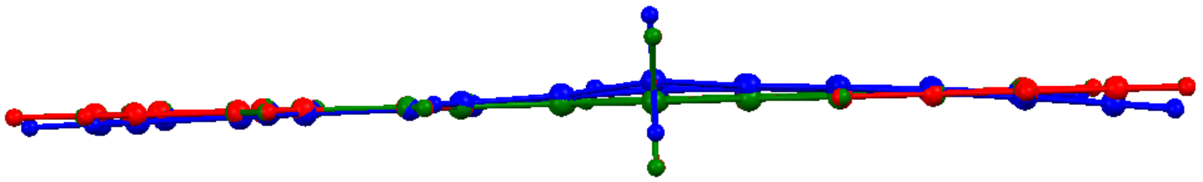

(b)

(c)

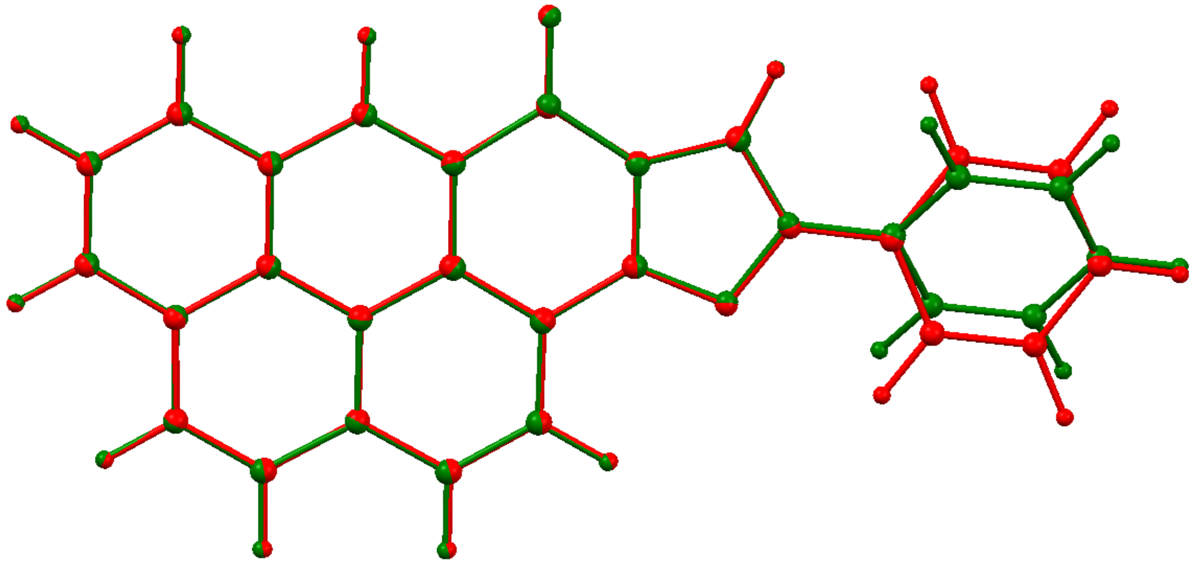

(d)

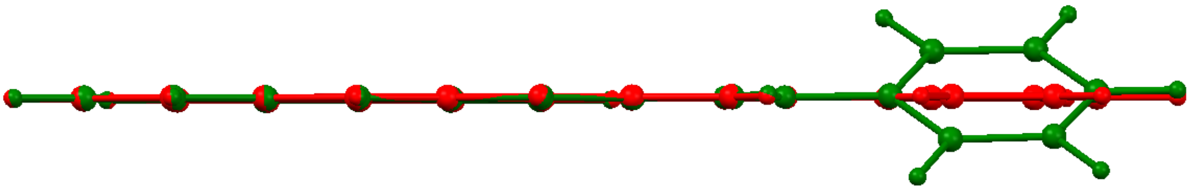

Figure S2. The overlay of the molecular structures of **3** (a) perpendicular to pyrene plane (b) along the C14 – C1 bond and **4** (c) perpendicular to pyrene plane and (d) along the C14 – C1 bond, obtained by: geometry optimization (green), and geometry predicted for the first excited state by TD-DFT calculations (red). The structure obtained by X-ray diffraction (**3** molecule 2) presented in blue. The RMS deviations between the optimized structure and an average experimental geometry of **3** is ~0.11Å, the RMS deviation between the optimized structures of ground and the first excited state is ~0.04Å

Table S2. Comparison of experimental and theoretical geometries of **3** and **4**. Interatomic distances in Å for compound **3** are averaged over the independent molecules *1*, *2* and *3* (depicted in red, blue and green accordingly in Figure 7 of the main text), with standard deviations in parentheses. Enhanced are the interatomic distances which upon excitation undergo changes exceeding 10x experimental uncertainty of **3**, considered as the most significant.

**3**					**4**	
Interatomic distance Experimental average			GS	ES	GS	ES
			Theoretical Calculations		Theoretical Calculations	
N1 N2	1.364	(1)	1.3768	1.3843	1.3565	1.3556
N2 C17	1.337	(1)	**1.3488**	**1.3659**	**1.3275**	**1.3489**
C17 C18	1.407	(1)	**1.4226**	**1.4434**	1.4181	1.4239
C18 C19	1.371	(4)	1.3819	1.3768	1.3832	1.3742
C19 N1	1.370	(2)	1.3831	1.3890	**1.3513**	**1.4165**
N1 C20	1.421	(2)	1.4199	1.4073	**1.4203**	**1.3779**
C17 C1	1.454	(1)	**1.4520**	**1.4189**	**1.4485**	**1.4166**
C18 C26	1.495	(2)	1.4952	1.4890	1.4512	1.4463
C26 C3	1.518	(2)	1.5253	1.5155	1.4935	1.4832
C3 C2	1.451	(2)	**1.4530**	**1.4235**	1.4458	1.4371
C2 C1	1.416	(3)	**1.4211**	**1.4546**	**1.4107**	**1.4384**
C26 O1					**1.2282**	**1.2434**
C19-N1-C20-C25	0.3	(30)	**0**	**0**	**-29.68**	**0**

3. ^{1}H and ^{13}C NMR spectra of 3 and 4

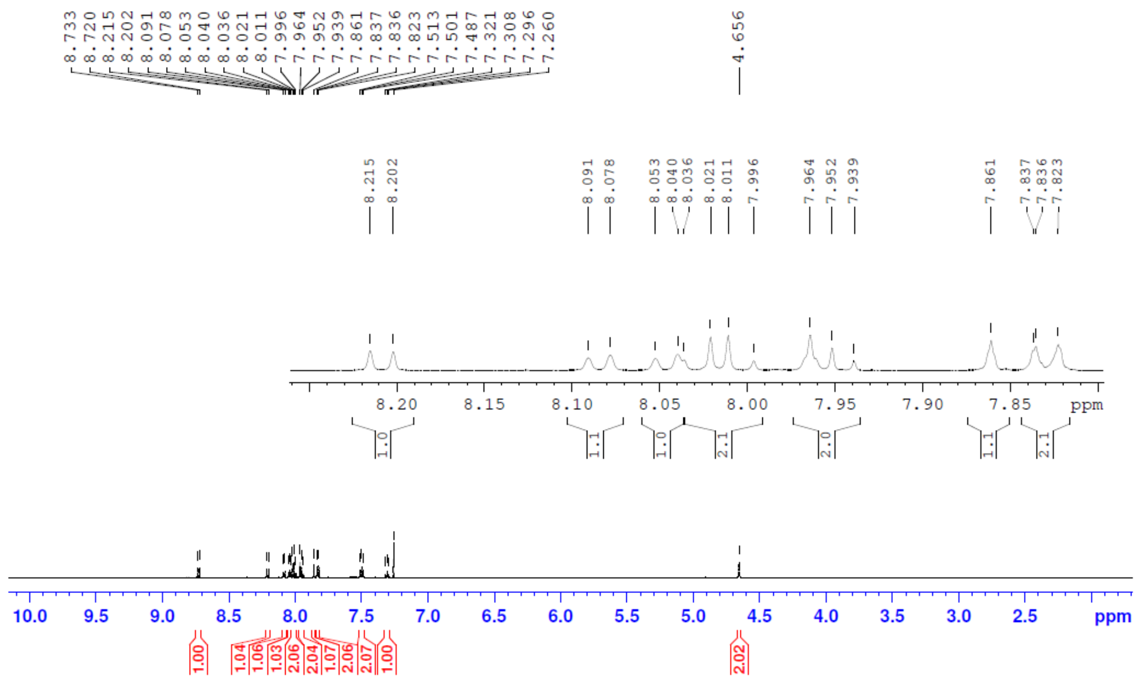

Figure S3. ^{1}H NMR spectrum of compound **3** (600 MHz, $CDCl_3$, room temperature).

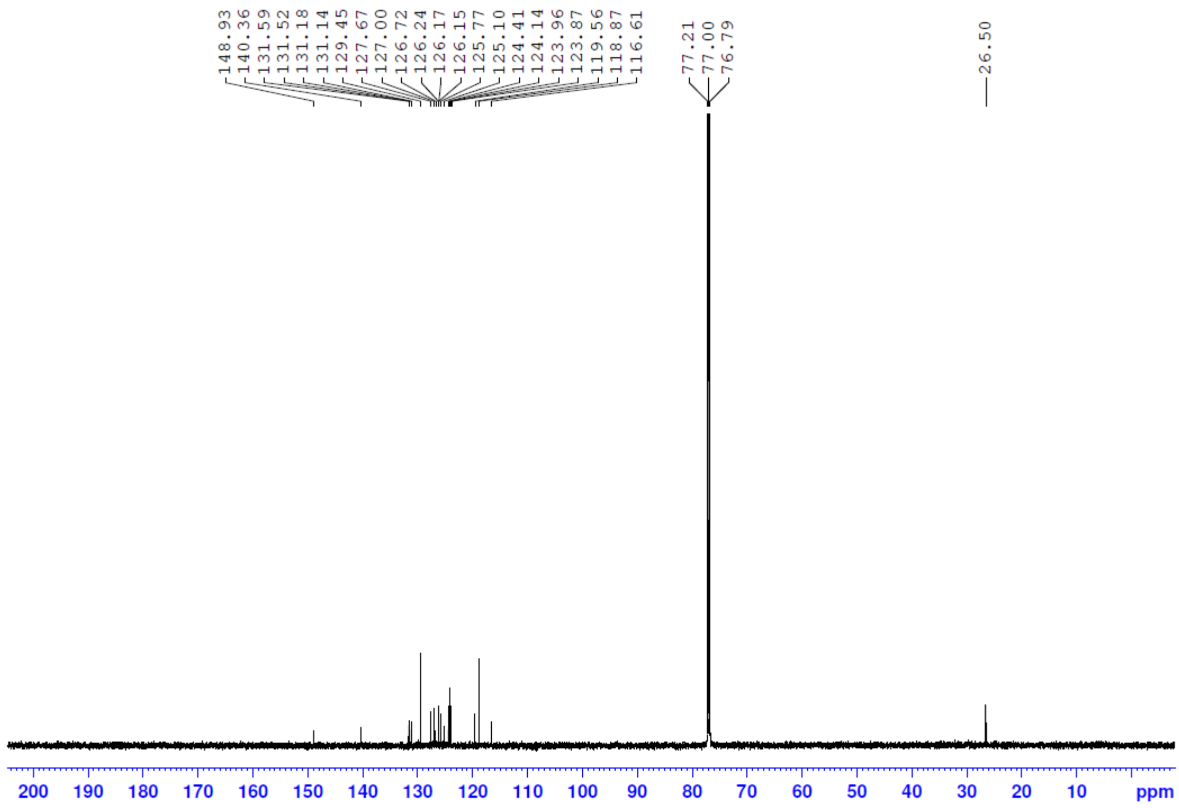

Figure S4. ^{13}C NMR spectrum of compound **3** (150 MHz, $CDCl_3$, room temperature).

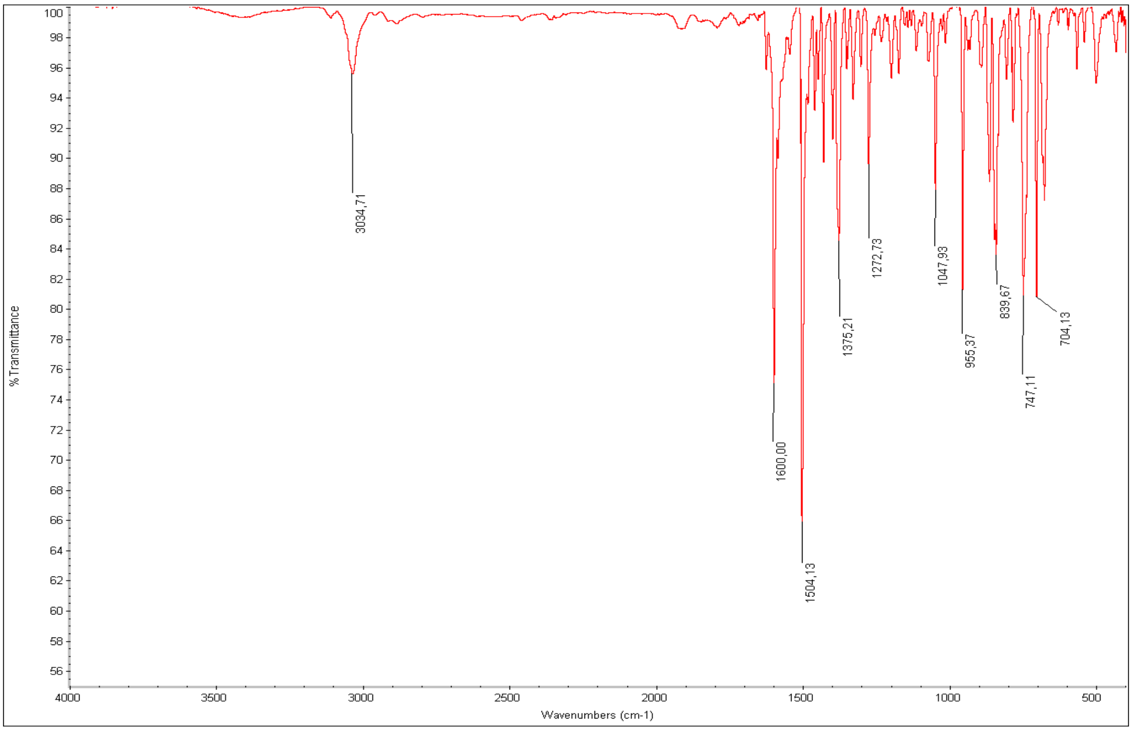

Figure S5. IR (KBr) spectrum of compound **3**.

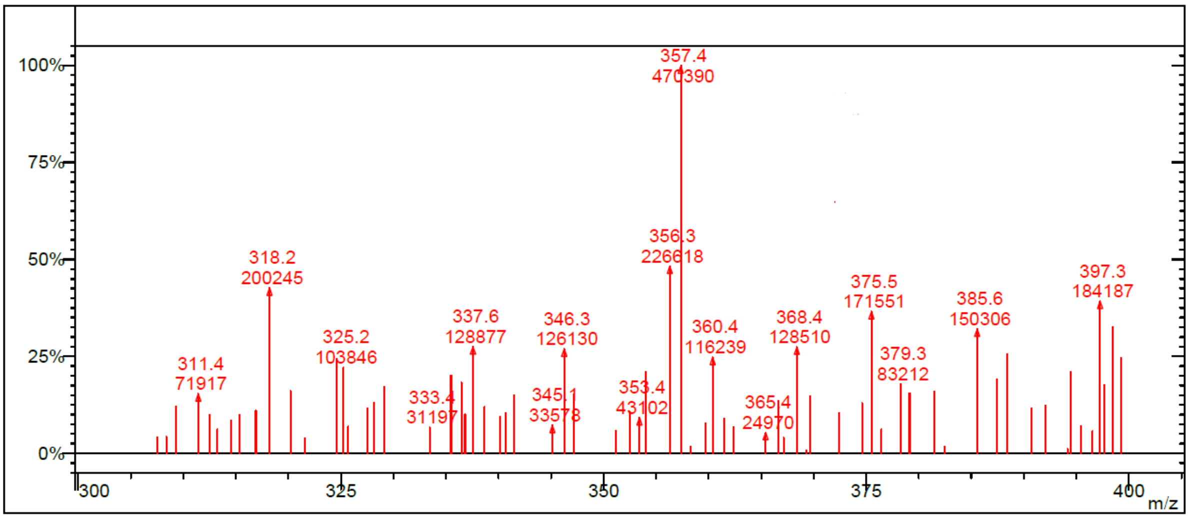

Figure S6. ESI MS spectrum of compound **3**.

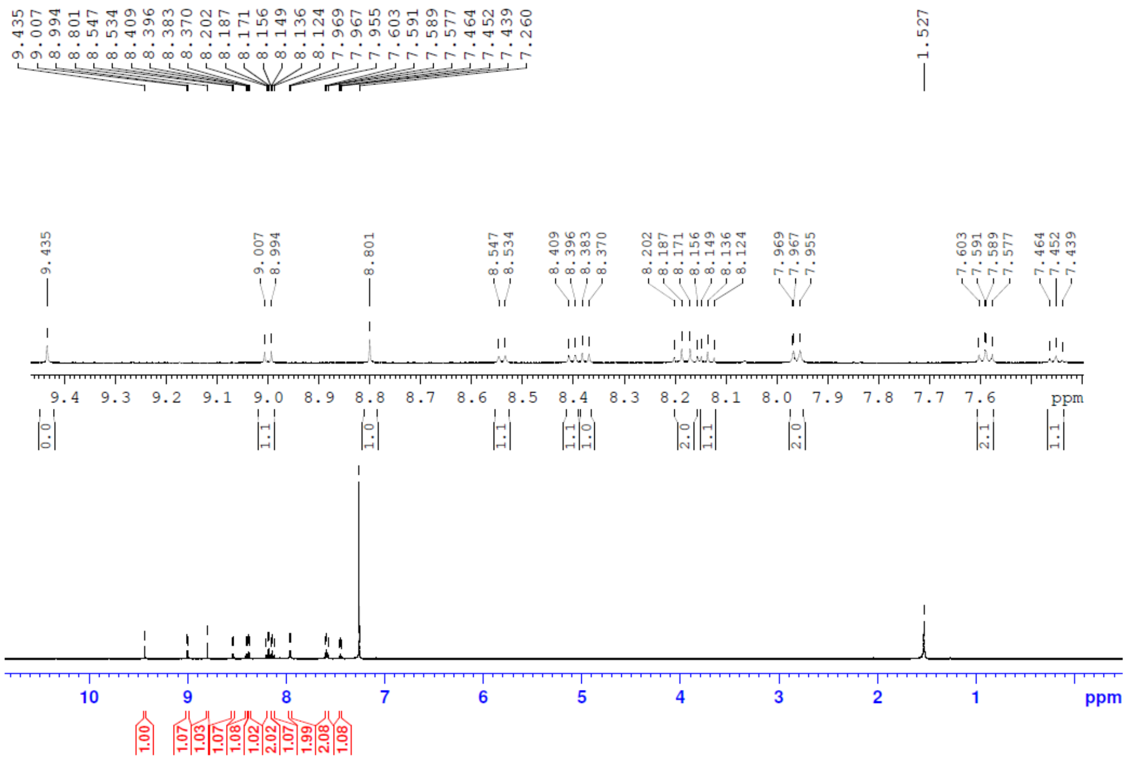

Figure S7. ^{1}H NMR spectrum of compound **4** (600 MHz, $CDCl_3$, room temperature).

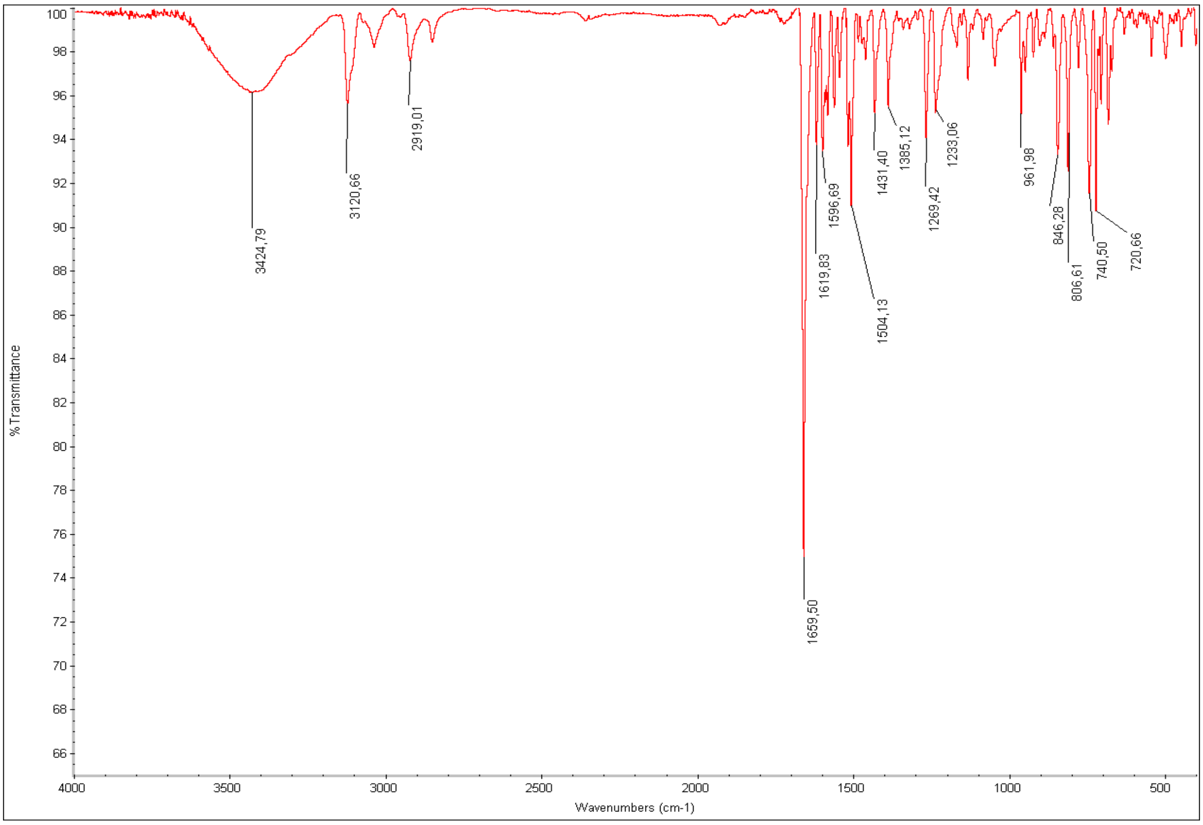

Figure S8. IR (KBr) spectrum of compound **4**.

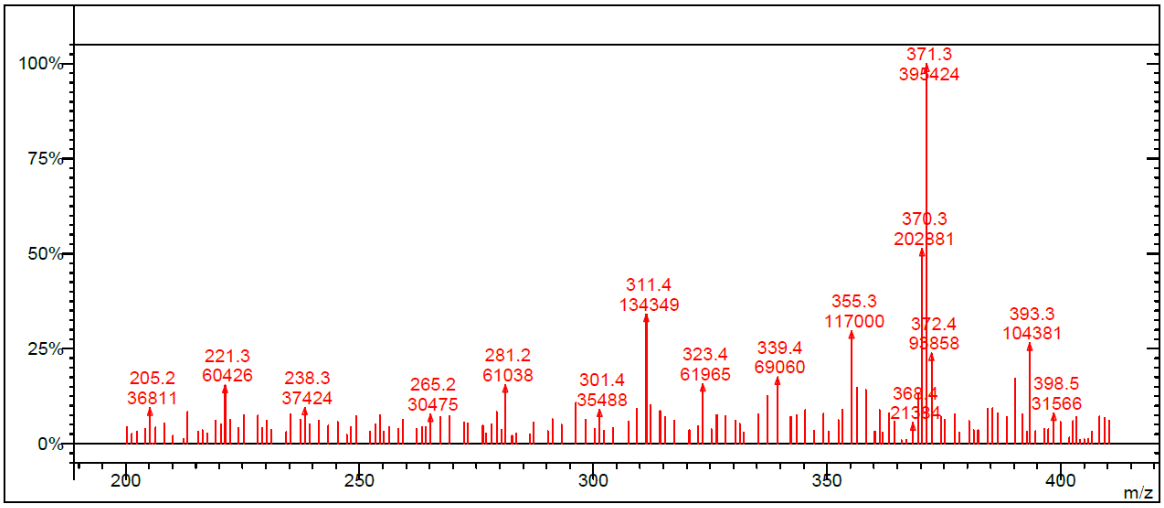

Figure S9. ESI MS spectrum of compound **4**.
